# Morphological comparison of internal auditory canal diverticula in the presence and absence of otospongiosis on computed tomography and their impact on patterns of hearing loss

**DOI:** 10.1007/s00234-020-02606-6

**Published:** 2020-11-18

**Authors:** Christian Burd, Irumee Pai, Melisha Pinto, Cristina Dudau, Steve Connor

**Affiliations:** 1 Department of Radiology, Guy’s and St Thomas’ NHS Foundation Trust, London, UK; 2 Department of Otolaryngology, Guy’s and St. Thomas’ Hospital NHS Foundation Trust, London, UK; 3 School of Biomedical Engineering & Imaging Sciences Clinical Academic Group, King’s College London, London, SE1 9RT UK; 4 Department of Oral Medicine and Radiology, Coorg Institute of Dental Sciences, Virajpet, Karnataka India; 5 Department of Neuroradiology, King’s College Hospital NHS Foundation Trust, London, UK

**Keywords:** Otosclerosis, Hearing loss, Diverticulum, Temporal bone, Tomography, X-ray computed

## Abstract

**Purpose:**

The association of internal auditory canal (IAC) fundal diverticula with otospongiosis (OS) and their clinical significance remain unclear. We explored whether isolated IAC diverticula were morphologically different from those with additional CT features of OS, and whether IAC diverticula morphology influenced patterns of hearing loss.

**Methods:**

Consecutive temporal bone CT studies with (*n* = 978) and without (*n* = 306) features of OS were retrospectively assessed. Two independent observers evaluated the presence of IAC diverticula morphological features (depth, neck:depth ratio, definition of contour and angulation of shape), and these were correlated with the presence of fenestral and pericochlear OS. Audiometric profiles were analysed for the isolated IAC diverticula and those with fenestral OS alone. Continuous data was compared using Wilcoxon rank sum tests and categorical data with chi-squared and Fisher’s exact tests.

**Results:**

Ninety-five isolated IAC diverticula were demonstrated in 54/978 patients (5.5%) without CT evidence of OS (31M, 23F, mean age 46), and 119 IAC diverticula were demonstrated in 71/306 patients (23%) with CT evidence of OS (23M, 48F, mean age 55). Reduced neck:depth ratio, ill definition and angulation were all significantly associated with the presence of pericochlear OS (*p* < 0.001), whilst only ill definition was associated with the presence of fenestral OS alone (*p* < 0.05). No morphological feature was associated with conductive hearing loss in isolated diverticula or with sensorineural hearing loss in diverticula with fenestral OS alone.

**Conclusion:**

IAC diverticula associated with pericochlear OS demonstrate different morphological features from isolated IAC diverticula. There are no clear audiometric implications of these morphological features.

## Introduction

**Fig. 1:**
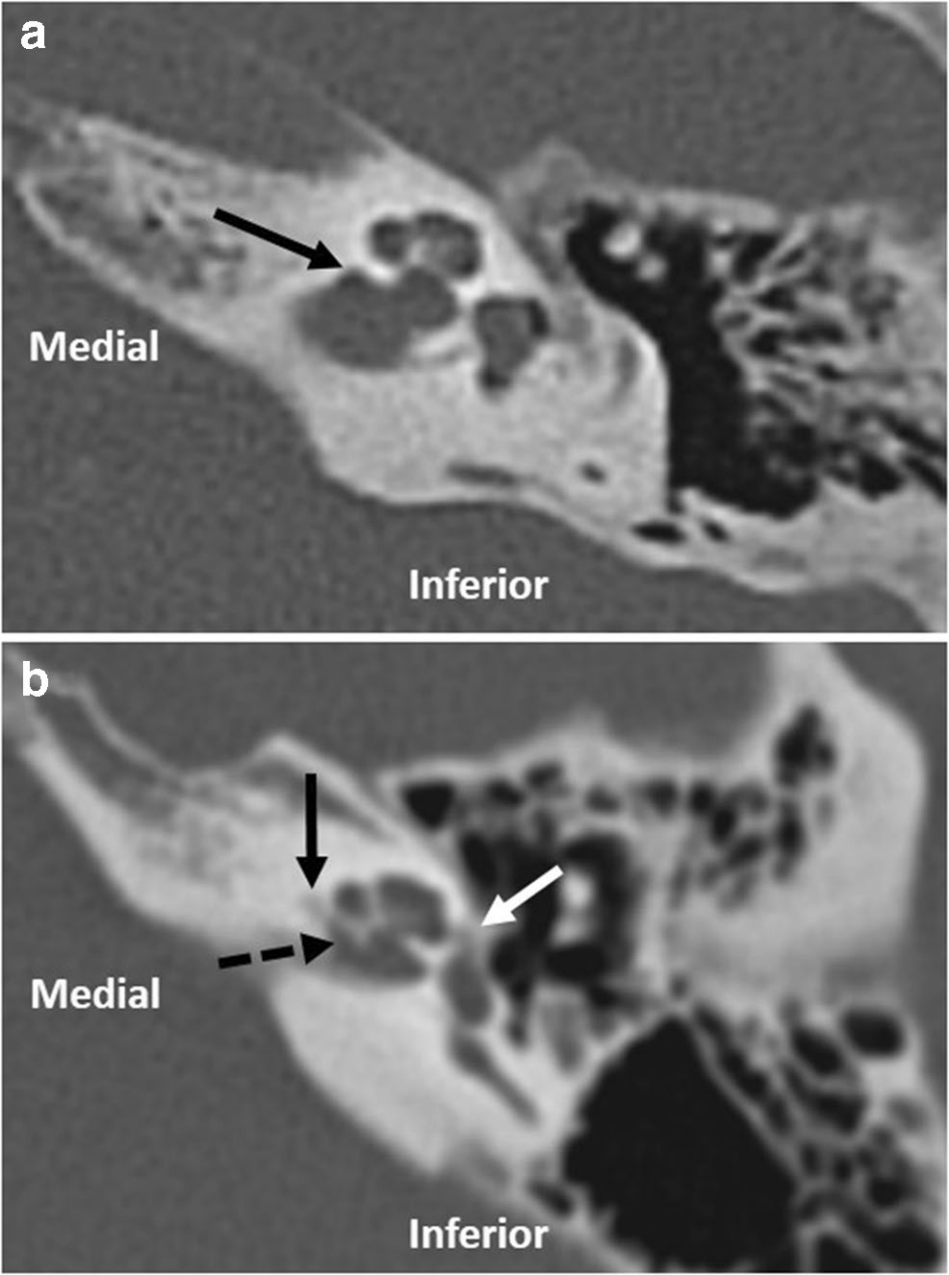
(**a**) Axial image demonstrating an isolated IAC diverticulum (black arrow). (**b**) Axial image demonstrating an IAC diverticulum (dashed black arrow) with concomitant pericochlear (solid black arrow) and fenestral (white arrow) lucency

Focal low attenuation diverticula have been demonstrated to arise from the antero-inferior margin of the internal auditory canal (IAC) fundus on CT [1–5]. Isolated IAC diverticula are seen in approximately 5% of temporal bone CT studies but their clinical significance remains uncertain [5, 6]. Such IAC diverticula are also described in the context of cavitary otospongiosis (OS) [3, 4] where diverticula associated with cavitary plaques are typically demonstrated in a similar location at the fundus of the IAC [4] (Fig. 1). Since the presence of these OS-related IAC diverticula appears to be independent of the extent of OS on CT, it is speculated that they may occur in isolation [4]. The clinical and pathogenic implication of isolated IAC diverticula and their association with OS therefore remains unclear.

It has been observed that IAC diverticula have a variable appearance on CT with respect to their depth, degree of definition and shape. Mihal et al. showed that the depth of the IAC diverticula differed between patients with and without OS [6] whilst Muelleman et al. found no relationship between diverticulum size and hearing loss [7]; however, the full implications of the different morphological characteristics is yet to be explored. We therefore aimed to ascertain whether there were particular morphological features of IAC diverticula which were associated with the presence of additional CT features of fenestral or pericochlear OS, and whether the morphology of the IAC diverticula influenced patterns of hearing loss in patients with or without OS. If demonstrated, then such CT features may have pathophysiological significance, and may be a useful diagnostic CT feature to highlight in the context of an isolated IAC diverticulum.

## Material and methods

This was a retrospective study performed in a tertiary centre and was approved by the institutional review board without the requirement for informed consent.

Two separate searches of the radiology information database were performed (CRIS, Healthcare software solutions ltd, Mansfield, UK). The first search, which identified cases without OS, collated consecutive CT temporal bone studies performed over a 2-year period between 2016 and 2018; those with radiological evidence of OS in either temporal bone were excluded on the basis of the report and subsequent imaging review. The second search, which identified cases with OS, collated CT temporal bone studies performed over a 6-year period between 2011 and 2017; those containing the terms “otosclerosis” or “otospongiosis” in the report or request text were further assessed to confirm the diagnosis of OS. Where there was CT evidence of OS, images were evaluated for the presence of both fenestral (“fenestral OS”) and pericochlear disease (“pericochlear OS”). All grades of OS in the Symons/Fanning classification were included in the study, with the group of pericochlear OS patients corresponding to Symons/Fanning classification grades 2 and 3 [8].

CT studies/patients with intrinsic bone disease, prior labyrinthine surgery or petrous bone trauma on the basis of the clinical request and report were excluded from either search.

86.5% of search 1 and 86.9% of search 2 of high-resolution CT temporal bone studies were performed on a single scanner (Philips Brilliance 40, Philips, the Netherlands; mA 100, kV 120, FOV 180 mm, matrix 768 × 768, pitch 0.348, slice thickness 0.67 mm, reconstruction index 0.33 mm). CT temporal bone studies on additional scanners were excluded if a slice thickness of <= 1 mm was not available.

The CT studies identified from the two searches were then reviewed for the presence of IAC diverticula by a head and neck radiologist. Multiplanar reformats parallel and perpendicular to the lateral semicircular canal were assessed on a high-resolution PACS workstation (Sectra workstation, Sectra AB, Linkoping, Sweden). IAC diverticula were defined as focal, nonvascular, low-density projections from the normal contour of the anterior or inferior IAC fundal wall, as identified on both axial and coronal images. There was no formal assessment of the Hounsfield units of the low-density projection but it was of similar density to the CSF at the IAC fundus when viewed with wide window widths. There was no minimum depth threshold.

### IAC diverticula analysis

CT studies in which IAC diverticula were present underwent a subsequent analysis to define the morphological features of the diverticula, using double sagittal oblique images reformatted in the plane of the IAC (Fig. 2). The images underwent standardised magnification and were viewed with wide window widths (400:4000). One head and neck radiologist, with 5 years of radiology experience, analysed the diverticula. Subsequently, a second head and neck radiologist, with 4.5 years radiology experience, separately analysed the diverticula. The isolated diverticula and diverticula with OS cases were analysed in a random order and blinded to the report. Where there was disagreement, the outcome was resolved by consensus.

**Fig. 2:**
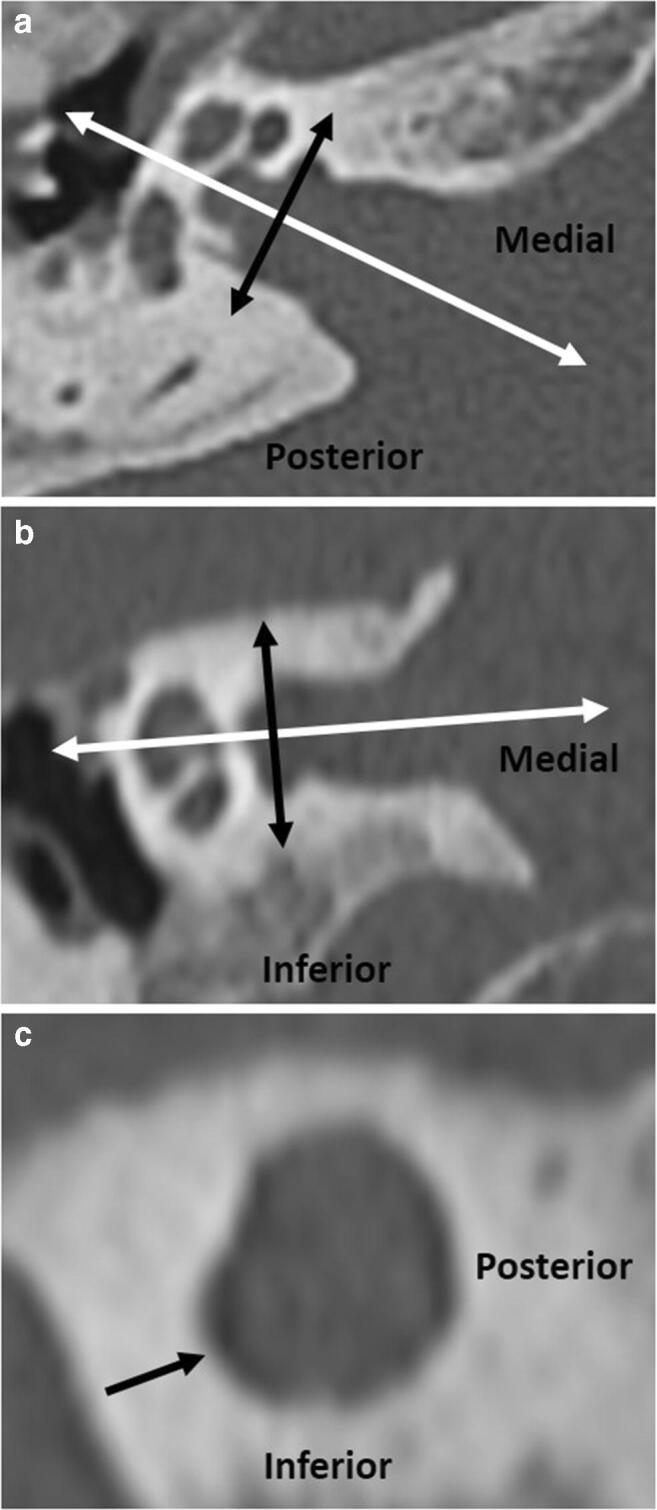
Double sagittal oblique images were obtained by reformatting the coronal and axial planes, on axial (**a**, white arrow) and coronal images (**b**, white arrow) respectively, in line with the IAC. This gives double sagittal oblique images, passing through the plane of the IAC diverticulum (black arrows in images **a**, **b** and **c**)

Morphological imaging features were partly based on those investigated in previous reports [6, 7] and for their potential ease of assessment in clinical reporting. The following morphological criteria were evaluated (Figs. 2 and 3):

- *Neck to depth ratio of diverticulum*: the ratio between the width at the base and the depth. A diverticulum is described as “broad” if the neck to depth ratio is > 1, and as “narrow” if ≤ 1.
- *Contour of diverticulum*: A qualitative assessment of the contour, termed as either “well-defined” (smooth regular contour similar to the adjacent IAC margin) or “ill-defined” (irregular contour in comparison to the adjacent normal linear smooth IAC margin).
- *Shape of diverticulum*: A qualitative assessment of the shape of the apex, termed as either “rounded” or “angulated”.

**Fig. 2:**
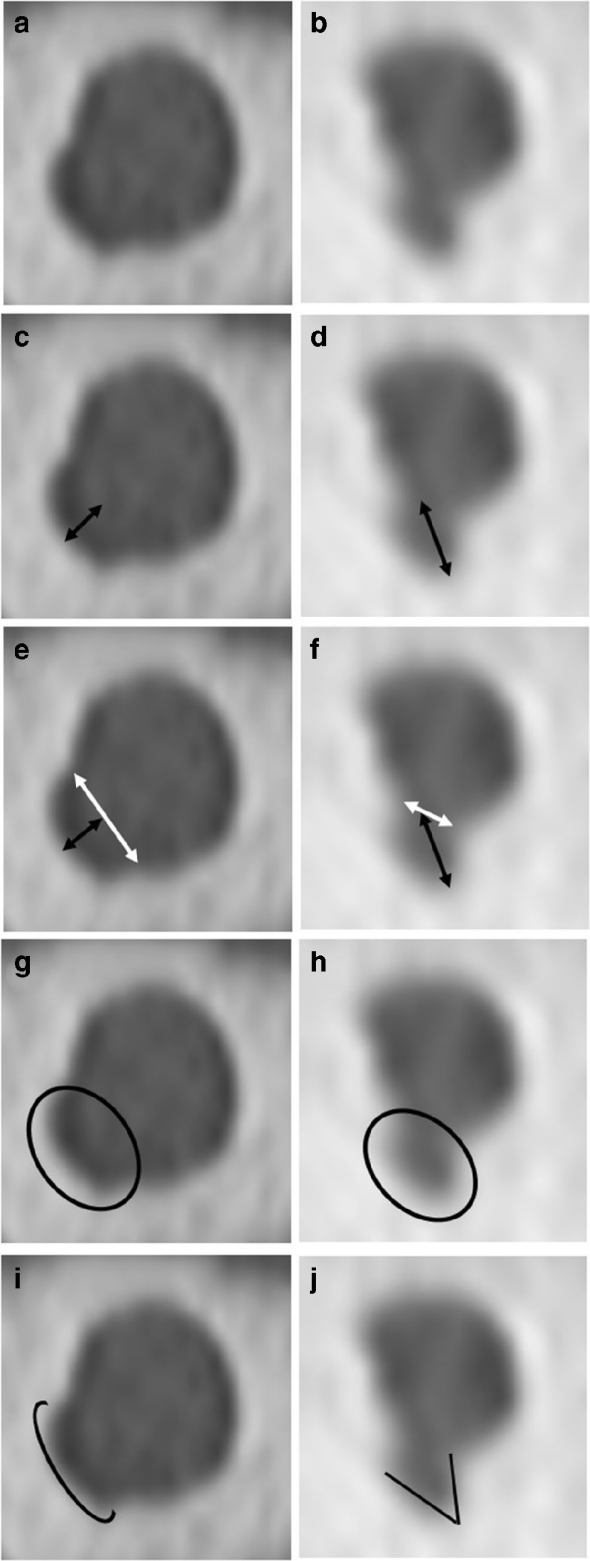
Double sagittal oblique images demonstrating an isolated diverticulum (**a**, **c**, **e**, **g**, **i**) and a diverticulum with concomitant OS changes (**b**, **d**, **f**, **h**, **j**) demonstrating morphological assessment. **c** and **d** Diverticulum depth measured from the IAC wall origin (black arrows). **e** and **f** Diverticulum neck measured by width at base (white arrows):depth (black arrows), with broad (**e**) and narrow (**f**) necks shown. **g** and **h** Diverticulum contour assessed as well (**g**) or ill-defined (**h**). **i** and **j** Diverticulum shape assessed at the apex for rounded (**i**) and angulated (**j**) morphology

### Hearing loss analysis

The association between IAC diverticula on imaging and hearing loss was also assessed. In order to evaluate the true impact of the presence of diverticula on hearing, audiometric analysis excluded cases where another significant potential cause existed for conductive hearing loss or mixed hearing loss (e.g. chronic otitis media, previous middle ear surgery) or sensorineural hearing loss (e.g. pericochlear OS, temporal bone fracture involving the otic capsule, labyrinthitis ossificans).

The audiometric definitions of conductive (CHL), mixed (MHL) and sensorineural hearing loss (SNHL) are shown in Table 1.

**Table 1:** Definitions of different types of hearing loss

Type	Definition
Normal	AC thresholds ≤ 20dBHL between 0.25 and 8 kHz
Conductive	BC thresholds ≤ 20dBHL between 0.5 and 4 kHz, and ABG ≥ 15 dB at one or more frequencies between 0.5 and 2 kHz
Sensorineural hearing loss	AC thresholds > 20dBHL between 0.25 and 8 kHz, and ABG < 15 dB between 0.5 and 2 kHz
Mixed	BC thresholds > 20dBHL between 0.5 and 4 kHz, and ABG ≥ 15 dB in at least one of the frequencies between 0.5 and 2 kHz

*AC*, air conduction; *BC*, bone conduction; *ABG*, air-bone gap

### Statistical analysis

The patient age was compared between the two groups using a *T* test. The presence of bilateral diverticula and the qualitative IAC diverticula neck, contour and shape criteria were assessed for association with the presence or absence of CT features of OS using a chi-squared test. The sensitivity, specificity and positive and negative predictive values for the presence of additional CT features of OS were calculated for each individual and combined morphological CT criteria. The association between type of hearing loss and diverticula morphology was assessed using chi-squared and Fisher’s exact tests. Statistical significance was set at *p* < 0.05. Cohen’s kappa (for categorical data) and intraclass correlation coefficient (ICC) were used to calculate inter-observer agreement.

## Results

### Descriptive statistics

The first search identified 978 CT studies with no evidence of OS and the second search 306 studies with OS on CT. From the first search, there were 54/978 (5.5%) CT studies which demonstrated isolated IAC diverticula. Of these 54 patients (31 male, 23 female, a mean age of 46 and an age range of 4–93 years), 13 were unilateral (24.1%) and 41 were bilateral (75.9%). There were therefore a total of 95 isolated IAC diverticula cases for analysis. From the second search, there were 71/306 (23%) CT studies which demonstrated IAC diverticula with OS. Of these 71 patients (23 male, 48 female, a mean age of 55 and an age range of 26–82 years), 23 were unilateral (32%) and 48 were bilateral (68%). There were therefore a total of 119 IAC diverticula cases with OS for analysis. Of these, 44 ears had fenestral OS only (37%) and 75 had additional pericochlear OS (63%). All ears with pericochlear OS had co-existent fenestral OS. In the isolated IAC diverticula group (*n* = 95), the mean diverticula depth was 1.1 ± 0.4 mm, compared to 1.8 ± 1 mm in the diverticula with OS group.

There was no significant difference between the two groups in patient age (*p* = 0.1) or the presence of bilateral diverticula (*p* = 0.3).

### IAC diverticula morphological assessment

The distribution of IAC diverticula neck configurations, definition and shape is detailed in Table 2. IAC diverticula CT features of a reduced neck to depth ratio, ill definition and angulated configuration were all significantly associated with the presence of pericochlear OS (*p* < 0.001), whilst only ill-defined IAC diverticula were associated with the presence of fenestral OS alone (*p* < 0.05) (Table 2).

**Table 2:** Comparison of the number and morphological characteristics of IAC diverticula in patients with and without CT features of otospongiosis

Diverticula morphological features	Isolated diverticula (*N* = 95)	Diverticula with OS (*N* = 119)	*p* value (isolated diverticula vs diverticula with OS)	Fenestral (*N* = 44)	*p* value (isolated diverticula v diverticula with fenestral OS alone)	Pericochlear (*N* = 75)	*p* value (isolated diverticula vs diverticula with pericochlear OS)
Broad/narrow neck (%)	87/8 (91.6/8.4%)	58/61 (48.7/51.3%)	< 0.001	37/7 (84.1/15.9%)	0.303	21/54 (28.0/72%)	< 0.001
Well/ill-defined (%)	82/13 (86.3/13.7%)	39/80 (32.8/67.2%)	< 0.001	30/14 (68.2/31.8%)	0.022	9/66 (12.0/88.0%)	< 0.001
Rounded/angulated (%)	82/13 (86.3/13.7%)	70/49 (58.8/41.2%)	< 0.001	37/7 (84.1/15.9%)	0.930	33/42 (44.0/56%)	< 0.001
Broad, well-defined, rounded/narrow, ill-defined, pointed (%)	76/6 (80.0/6.3%)	35/41 (29.4/34.5%)	< 0.001	27/4 (61.4/9.1%)	0.574	8/37 (10.7/49.3%)	< 0.001

The sensitivity, specificity, PPV and NPV for significant IAC diverticula morphological features with respect to the presence of pericochlear and fenestral OS are demonstrated in Table 3.

**Table 3:** Comparison of the sensitivity, specificity and positive and negative predictive values for the presence of diverticula morphological features associated with OS (*p* < 0.05) and the concomitant presence of fenestral and pericochlear otospongiosis on CT

OS group	Morphological features	Sensitivity (95% CI)	Specificity (95% CI)	PPV (95% CI)	NPV (95% CI)
Fenestral	Ill definition	31.82% (18.61–47.58%)	86.32% (77.74–92.51%)	51.85% (35.64–67.68%)	73.21% (68.75–77.25%)
Pericochlear	Ill definition	88.00% (78.44–92.51%)	86.32% (77.74–92.51%)	83.54% (75.26–89.44%)	90.11% (83.08–94.41%)
	Narrow neck	72.00% (60.44–81.76%)	91.58% (84.08–96.29%)	87.10% (77.41–93.01%)	80.56% (74.14–85.69%)
	Angulated configuration	56.00% (44.06–67.45%)	86.32% (77.74–92.51%)	76.36% (65.23–84.76%)	71.30% (65.53–76.46%)

*OS*. otospongiosis; *PPV*, positive predictive value; *NPV*, negative predictive value; *CI*, confidence interval

### Inter-observer agreement

ICC analysis for diverticula depth was 0.92. Cohen’s kappa analysis of inter-observer agreement for neck configuration was 0.91 (near perfect agreement) and 0.80 for both contour definition and shape scoring (substantial agreement). Upon subsequent joint observer review, an agreed consensus was achieved in all cases.

### Hearing analysis

In the isolated IAC diverticula group, there were 30 ears without another significant potential cause for CHL or MHL on the CT study. The audiometric profiles in this group were normal hearing 18 (60%), SNHL 8 (27%), MHL 3 (10%) and CHL 1 (3%). There was no correlation between the type of hearing loss and any of the morphological features (*p* < 0.05).

In the IAC diverticula with OS group, there were 38 ears with fenestral OS alone and no other significant potential cause for SNHL or MHL. The audiometric profiles were normal hearing 9 (24%), SNHL 7 (18%), MHL 14 (37%) and CHL 8 (21%). There was no correlation between the type of hearing loss and any of the morphological features (*p* < 0.05). Of the 38 ears in this group, the IAC diverticulum was a unilateral finding in 10 ears; in all 10, the underlying cochlear thresholds were symmetric, so there was no difference in the inner ear hearing function between the two sides.

## Discussion

Our results indicate that qualitative IAC diverticula CT criteria of a reduced neck to depth ratio, ill definition and angulated configuration are significantly associated with the presence of pericochlear OS, whilst an ill-defined IAC diverticulum is significantly associated with the presence of fenestral OS alone. However, we have observed no correlation between IAC diverticula morphology and the presence CHL with isolated diverticula, or SNHL/MHL in patients with fenestral OS alone.

There remains lack of clarity over the implication of IAC diverticula in patients with and without OS. Our finding of a 5.5% prevalence for isolated IAC diverticula on CT is in keeping with previous studies [5, 6]. We also demonstrated IAC diverticula to be present in 23% of patients with additional CT features of OS, as compared with 10–39% incidence of cavitary OS at this site in previous studies [3, 4, 9–11]. There remains no clear consensus on the clinical significance of isolated IAC diverticula and their potential relation to the cavitary plaques of OS [4–6], with these two terms being used interchangeably to describe a similar entity. Since there has been shown to be no correlation of such cavitary plaques at the fundus of the IAC with the extent of OS, it appears possible that they could occur in isolation and thus be potentially indistinguishable from the isolated IAC diverticula [4].

We explored whether there were morphological features of the IAC diverticula on CT which enabled a distinction of an isolated IAC diverticulum from that associated with fenestral or pericochlear OS. It could be speculated that the strong correlation between specific morphological characteristics of IAC diverticula and the presence of other CT features of OS would imply that an isolated IAC diverticula with these features would more likely be associated with OS. Mihal et al. [6] measured the depth of diverticula in a smaller group of patients with IAC diverticula, and concurred that they were larger in patients with CT features of OS. However, to our knowledge, this is the first study focused on comparing specific morphological features of IAC diverticula with and without CT findings of OS.

The aetiology of IAC diverticula remains uncertain; however, their location has been proposed to correlate with the site of transition of primary enchondral bone to secondary bone formation and the persistence of cartilaginous rests [8, 12]. It may be hypothesised that the isolated diverticulum represents a normal developmental variant, a distinct pathological process such as a form of cavitary OS, or possibly a pre-existing IAC diverticula acting as a nidus for OS. The morphological features of reduced neck to depth ratio, ill definition and angulated configuration occurred much more frequently in cases with pericochlear OS than with isolated fenestral OS, which argues that they may correspond to retro-fenestral OS superimposed on a pre-existing diverticula variant, or alternatively a primary form of cavitary fundal OS in conjunction with pericochlear OS. It is of interest that the incidence of pericochlear extension in patients with IAC diverticula and OS (26.4%) far exceeded that demonstrated on CT in OS overall (3.2%) which suggests the latter to be more likely.

When the radiologist observes an IAC diverticulum, its implications may therefore depend on the morphological features. In particular, it may direct the search for CT features of otospongiosis which may occasionally be equivocal in terms of diagnosis and extent. Firstly, it has been demonstrated that diverticula with reduced neck to depth ratio, ill definition and angulated configuration are strongly associated with CT overt pericochlear OS and such diverticula features should prompt a careful inspection for associated imaging features. Both a narrow neck and ill-defined morphology had a PPV value > 80% for pericochlear OS. However, the CT finding of pericochlear OS is usually associated with reduced bone conduction thresholds [13]; and since SNHL and MHL were not more frequent in patients with diverticula demonstrating these morphological features and fenestral OS only, this argues against the potential for sub-radiologic pericochlear OS. Interestingly, normal bone conduction has also been demonstrated in a previous study of cavitary IAC fundal plaques/diverticula in OS [4]. Secondly, the implications of an isolated ill-defined IAC diverticulum are also intriguing and its role in diagnosing occult sub-radiologic otospongiosis should be explored. The finding of an ill-defined diverticulum demonstrated some specificity (83%) for the presence of fenestral otospongiosis and was rarely an isolated feature. However, whilst it may be postulated that the diverticulum represents the earliest manifestation of OS on CT, and that the stapes footplate and fissula ante fenestram should be carefully inspected for subtle CT features of OS, the lack of association we demonstrated with CHL/MHL would argue against this.

Our retrospective study has several potential limitations. Firstly, since the CT features of OS were evident on the CT studies, it was not possible to blind the observers as to the groups of patients with and without features of OS. Secondly, the inclusion of studies from more than one CT scanner means there is potentially inherent bias secondary to variations in technical factors, for example poorer quality acquisition could bias the evaluation of IAC diverticula shape and definition; however, > 85% of CT scans were performed on a single scanner in both groups. Thirdly, it should be appreciated that the presence of CT findings of OS may not be a sufficient reference standard. Subclinical OS may be present in up to 11% of the population histologically and may not be evident on imaging [14]. Therefore, it is possible that OS is actually present in our group of patients with isolated diverticula, and this is a potential explanation for the presence of some patients with typical morphological appearances of OS-related diverticula despite no additional CT evidence of OS. Finally, since middle ear disease and pericochlear OS were prevalent in our study groups, this limited the number of subjects within our study groups which could be used to provide meaningful correlation with audiometric outcomes.

There is scope for future clarification of these findings. It would be interesting to evaluate other imaging features such as the objective assessment of Hounsfield unit or MRI evidence of CSF signal for their ability to distinguish patients with OS. Since the depth and morphology of the diverticula may evolve, whilst the CT and clinical features of OS become more evident with time, a longitudinal study would also be of benefit. This would be of particular interest in the case of isolated IAC diverticula with morphological features we have shown to be associated OS.

## Conclusions

It is recommended that a reduced neck to depth ratio, ill definition and angulated configuration morphological features of IAC diverticula should prompt careful inspection for other CT features of OS, and particularly pericochlear OS. There are no clear audiometric implications of these IAC diverticula morphological features.
